# Theoretical models of synaptic short term plasticity

**DOI:** 10.3389/fncom.2013.00045

**Published:** 2013-04-19

**Authors:** Matthias H. Hennig

**Affiliations:** School of Informatics, Institute for Adaptive and Neural Computation, University of EdinburghEdinburgh, UK

**Keywords:** short term plasticity, synaptic transmission, mathematical model, synaptic depression, synaptic facilitation

## Abstract

Short term plasticity is a highly abundant form of rapid, activity-dependent modulation of synaptic efficacy. A shared set of mechanisms can cause both depression and enhancement of the postsynaptic response at different synapses, with important consequences for information processing. Mathematical models have been extensively used to study the mechanisms and roles of short term plasticity. This review provides an overview of existing models and their biological basis, and of their main properties. Special attention will be given to slow processes such as calcium channel inactivation and the effect of activation of presynaptic autoreceptors.

## Introduction

Chemical synapses are highly specialized structures that enable neurons to exchange signals, or to send signals to non-neural cells such as muscle fibers. Even though there is a staggering diversity of synapse morphologies and types in the brain, the fundamental process of synaptic transmission is always the same. A presynaptic membrane potential depolarization, typically caused by the arrival of an action potential, triggers the release of neurotransmitter, which then binds to receptors that, in turn, generate a response in the postsynaptic neuron.

A key quantity in neural circuits is the synaptic efficacy or strength, which varies over time. Cellular processes such as long-term potentiation and depression contribute to the patterning of the nervous system during development, and are thought to constitute the basis of learning and memory (Morris, 2003). Slow and long-lasting homeostatic processes adjust synaptic strength to maintain circuit activity within functional regimes (Turrigiano and Nelson, 2004). In addition, a whole range of activity-dependent processes exist that modulate synaptic efficacy continuously on very short time scales ranging from milliseconds to minutes (for reviews, see Zucker and Regehr, 2002; Fioravante and Regehr, 2011). Unlike long-term and homeostatic plasticity, *short term plasticity*, the topic of this review, has a direct influence on the computation performed by neural circuits as these dynamics take place on the time scale of stimulus-driven activity, neural computations and behavior.

Broadly, short term plasticity can be classified as synaptic depression and facilitation. Depression refers to the progressive reduction of the postsynaptic response during repetitive presynaptic activity, while facilitation is an increase synaptic efficacy. Each of these may be caused by a range of different mechanisms with different time constants, and the two forms are not mutually exclusive. For instance, a particularly well-studied example of a strongly depressing synapse is the calyx of Held, a giant synaptic terminal in the mammalian auditory brainstem (Schneggenburger and Forsythe, 2006). A closer look at the underlying mechanisms, however, reveals that the response is also modulated by facilitation, which is however, partially masked by depression. In fact, most synapses express some combination of these two mechanisms, but with considerable variability between different neuron types (Wang et al., 2006).

The purpose of this review is to summaries models of short term plasticity, to discuss their biological background and plausibility, and to provide a guide for selecting an appropriate model and level of detail. The focus here is on the mechanistic aspects of these models, for a review of functional implications see Abbott and Regehr (2004). The review begins with a reminder of the main processes involved in synaptic transmission. Next, the vesicle depletion model and its variants will be introduced as a canonical model for short term plasticity. Finally, several additions to this class of models will be discussed that were required to explain more recent experimental findings.

## Principles of synaptic transmission

**Figure 1:**
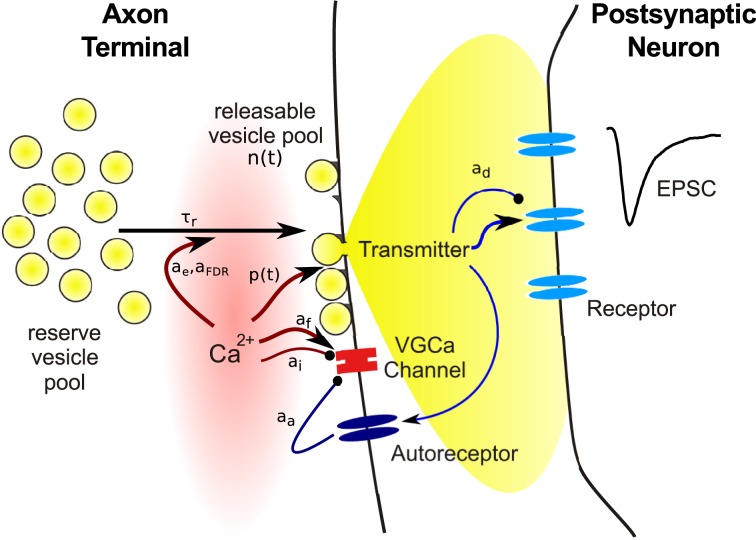
**Schematic illustration of the main steps involved in synaptic transmission, and of variables subject to use-dependent modification.** Symbols refer to quantities used in the model equations in this review.

Almost all factors contributing to short term plasticity are located in the presynaptic terminal. To identify the relevant variables required in models, we begin with a brief review of the main events following the arrival of a presynaptic action potential at a synapse, as illustrated in Figure 1. The site where synaptic transmission of neural activity is initiated is called the *active zone* (AZ), a presynaptic morphological specialization where vesicles containing neurotransmitter and proteins required for the release process are clustered. The AZ is opposed by the *postsynaptic density* (PSD), an area that contains a large number of different proteins implicated in synapse maintenance and plasticity. In addition to a whole variety of structural and signaling complexes, the PSD contains the bulk of the neurotransmitter receptors mediating the postsynaptic response.

Neurotransmitter release from vesicles located at the AZ is initiated by an elevation of the intracellular calcium concentration [Ca^2+^]_*i*_ due to opening of voltage gated calcium channels (VGCC). VGCCs are thought to be tightly co-localized with AZs, such that the arrival of the presynaptic action potential causes an increase of [Ca^2+^]_*i*_ within a localized nanodomain from around 30 nM at rest to about 10–30 μM. This brief elevation of [Ca^2+^]_*i*_ increases the probability of vesicle fusion with the cell membrane and subsequent release of transmitter into the synaptic cleft. Hence the release probability *p*(*t*) is the first variable required in a model of short term plasticity. Importantly, the relationship between [Ca^2+^]_*i*_ and release probability *p* is not linear, but follows a steep power function relationship with an exponent between three and four (Bollmann et al., 2000; Schneggenburger and Neher, 2000; Lou et al., 2005). The release probability is often modulated in an activity-dependent manner, hence it is expressed as a function of time.

Electronmicrographs show that presynaptic terminals contain vesicles filled with neurotransmitter. The release of a single vesicle then constitutes the smallest signal (or *quantum*) that can be transmitted to the postsynaptic neuron, which can be seen as spontaneous miniature postsynaptic current at an unstimulated synapse. Usually only a small fraction of the vesicles in the terminal are located in close vicinity of the cell membrane at the AZ. These vesicles are assumed to be release-ready or “primed,” while the remaining are assumed to be on hold to replace empty vesicles following transmitter release. The existence of anatomically distinguishable vesicle populations has led to the concept of *vesicle pools*: docked vesicles form the *releasable pool* and those in waiting the *reserve pool*. The release process is termed *excocytosis*, which is followed by the retrieval of empty vesicles through *endocytosis*, and *replenishment* of vesicles on available release sites from the reserve pool. There is evidence that more than two vesicle pools may exist, which differ in release probability and retrieval rate (Trommershäuser et al., 2003; Wölfel et al., 2007), which may be due to their distance from VGCCs (Wadel et al., 2007). However, the details of this matter are still debated and will not further discussed here (for reviews, see Sudhof, 2004; Rizzoli and Betz, 2005).

Hence the second variable required in a synapse model is the number of vesicles *N*(*t*) available for release. Again, as will be discussed in more detail below, the number of release-ready vesicles changes over time since the occupancy of the pool changes during neural activity. Vesicle number and release probability are the key ingredients for a model of presynaptic transmitter release:

(1) T(t)=p(t) · N(t)

Here *T*(*t*) is the amount of transmitter released into the synaptic cleft at time *t*. Simulating a highly realistic synapse model using this expression would require a precise, time continuous model of calcium influx and vesicle cycling. However, since the release probability dramatically increases upon the arrival of a presynaptic action potential from a resting value of almost zero, it is usually sufficient to update these quantities only once every time a presynaptic action potential arrives.

Finally, the released transmitter diffuses through the synaptic cleft and binds to receptors to generate a postsynaptic response, the main quantity of interest in synapse models. Here, we focus on the action of ionotropic receptors, which contain an ion channel that opens when transmitter is bound. The kinetics of such a response is determined by the rates of transmitter binding and unbinding and opening and closing of the channel, as well as transitions to and from desensitized states. The simplest model of this process is when the postsynaptic conductance is proportional to the amount of transmitter released:

(2) $$g(t)=g_{m}T(t)$$

The peak conductance is denoted by *g*_*m*_. If the time course of the response is relevant, for instance to distinguish between fast AMPA receptor and slow NMDA receptor mediated transmission, alpha functions, double exponential models, or simple kinetic models are useful to model this process (Destexhe et al., 1994b; Roth and Rossum, 2009).

Numerous studies have been devoted to assessing the release probability and quantal content of synapses in various brain areas and neurons types. As will be shown below, this is generally achieved through model-based analysis, which is possible because the synapse models provide a good mapping between experimental observables, usually the postsynaptic current and its variance, and the underlying synaptic parameters. A comprehensive overview of parameters of a range of neuron types assessed in this way can be found in a review by Branco and Staras (2009).

## The vesicle depletion model and extensions

### Vesicle depletion as main cause of synaptic depression

The outline in the preceding section hints that presynaptic vesicles are a limited resource, and that their depletion during ongoing activity can lead to a suppression of the postsynaptic response. The first formal model of such a process was published by Liley and North (1953), even before synaptic vesicles were discovered by De Robertis and Bennett (1955). It sought to explain synaptic depression during brief tetanic stimulation of the rat neuromuscular junction, and was based on the assumption that releasable neurotransmitter is produced at a limited rate. Tetanic stimulation was assumed to cause transmitter depletion and a concomitant reduction in postsynaptic response. This process is described by a simple first order kinetic model:

(3) $$\frac{dn(t)}{dt}=\underset{\text{replenishment}}{\underset{︸}{\frac{1-n(t)}{\tau_{r}}}}\underset{\text{release}}{\underset{︸}{-\sum_{j}\delta (t-t_{j})\;\cdot \;p\;\cdot \;n(t)}}$$

where *n*(*t*) is the occupancy of the release pool, bounded between zero and one, τ_*r*_ the time constant of the vesicle replenishment, and *t*_*j*_ the presynaptic spike times. Note that in this and all following equations, the dynamic quantity, here *n*(*t*), is evaluated before the delta function [as in *n*(*t* − ϵ), here the ϵ is omitted for clarity]. The release term reduces the vesicle pool occupancy by *T*(*t*) = *p* · *n*(*t*), which is proportional to the postsynaptic response (see Equation 2). Experiments suggest that the recovery time constant is typically in the order of seconds. Equation (3) describes a continuous form of the model, which may be inappropriate for synapses with a small number of releasable vesicles, as it is often the case. Then a discrete form should be used where the release pool occupancy *n*(*t*) is replaced by the vesicle number *N*(*t*). In this case, a discrete form is also required to accurately model the stochasticity of synapses.

**Figure 2:**
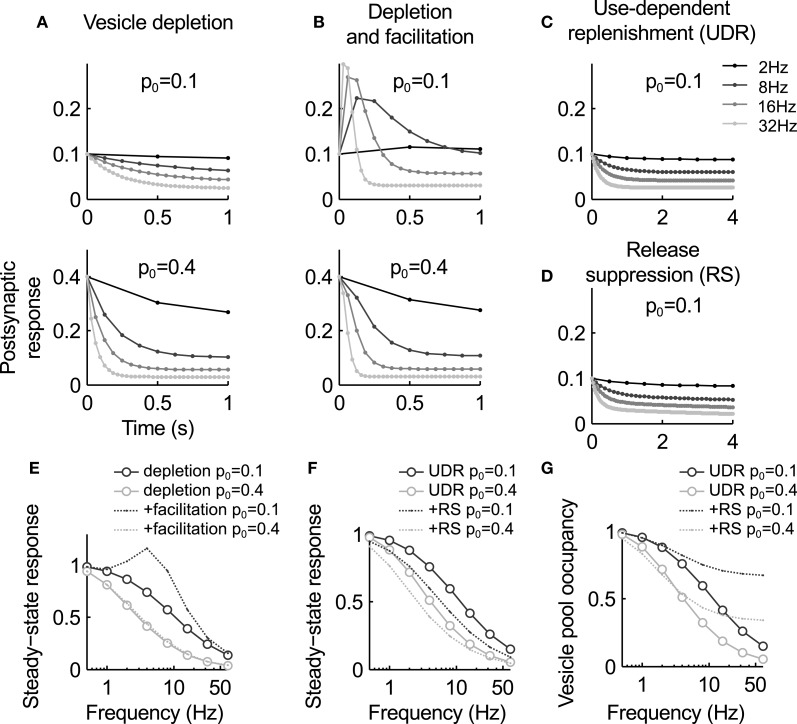
**Summary of the key characteristics of the models discussed in this review. (A–D)** Postsynaptic response for the different models during stimulation at different frequencies. **(A)** The vesicle depletion model (Equation 3) predicts exponential decay of the response and an inverse relation between stimulus frequency and steady-state amplitude. A higher release probability causes faster and stronger depression [compare upper and lower graph, see also panel **(E)**]. **(B)** The depletion model with facilitation (Equations 3, 4) predict a transient response increase during high-frequency stimulation. For a low basal release probability *p*_0_ the response remains elevated (top graph), while for higher *p*_0_ vesicle depletion masks facilitation [bottom graph, see also panel **(E)**]. **(C)** Use-dependent vesicle replenishment (Equation 6) increases the steady-state response. **(D)** As panel **(C)**, but with added slow use-dependent suppression of release probability. Here the postsynaptic response continues to slowly decay when the depletion model reaches steady-state [compare **(C)** and **(D)**]. **(E)** Steady-state response magnitude as a function of input frequency for the depletion model (circles) and the depletion model with facilitation (dashed lines). **(F)** Same as **(E)**, but for the depletion model with use-dependent replenishment (UDE, circles) and the UDE model with slow suppression of release probability (RS, dashed). Note that the latter increases depression in particular at low frequencies. **(G)** Occupancy of the releasable vesicle pool for the models in panel **(F)**. It is less depleted for the RS model as steady-state depression is mediated by the reduction in release probability. Parameters: τ_*r*_ = 1 s, *a*_*f*_ = 0.3, τ_*f*_ = 0.2 s [no facilitation in **(C,D)**], *a*_*e*_ = 0.4, τ_*e*_ = 0.1 s, *a*_*i*_ = 0.01, τ_*i*_ = 10 s.

This model predicts an exponential decay of the postsynaptic response during stimulation at a constant rate, and an inverse relation between input frequency ν and steady state level of depression *n*_ ∞_ = 1/(*p*ντ_*r*_ + 1) (Figures 2A,E). It was found to fit responses recorded from some depressing synapses very well (Liley and North, 1953; Tsodyks and Markram, 1997), including EPSCs during stimulation of the calyx of Held with *in vivo*-like activity patterns (Hermann et al., 2009). However, often synapses show substantial deviations. In particular, the steady state values decrease more slowly with increasing frequency than the inverse behavior predicted here.

### Synaptic facilitation

To explain such deviations from the deletion model, it was first suggested by Betz (1970) to extend it by release probability facilitation that counteracts depression. Potential underlying mechanism of facilitation are an accumulation of residual calcium in the synaptic terminal (Atluri and Regehr, 1996; Blatow et al., 2003; Felmy et al., 2003), which causes rapid VGCC facilitation (Katz and Miledi, 1968; Borst and Sakmann, 1998; Cuttle et al., 1998; Mochida et al., 2008). A simple phenomenological model of such processes is to increase the release probability after each presynaptic spike (Betz, 1970; Varela et al., 1997; Markram et al., 1998):

(4) $$\frac{dp(t)}{dt}=\frac{p_{0}-p(t)}{\tau_{f}}+\sum_{j}\delta (t-t_{j})\;\cdot \;a_{f}\;\cdot \;(1-p(t))$$

Here *p*_0_ is the baseline release probability, *a*_*f*_ the amount of facilitation per action potential and τ_*f*_ the recovery time constant. The time constant is typically in the range of tens of milliseconds, much faster than vesicle replenishment. Therefore, facilitation is usually observed during more intense periods of activity. Steady-state facilitation approaches *p*_∞_ = (*p*_0_ + ν*a*_*f*_τ_*f*_)/(1 + ν*a*_*f*_τ_*f*_) for a stimulus with constant frequency ν (Figure 2E).

The net effect of the combined model of facilitation and vesicle depletion depends strongly on the basal release probability: for a small *p*_0_, facilitation can have a substantial effect since it is not masked by rapid vesicle pool depletion, and for large values depression will dominate over depletion (Figure 2B). As a general rule, it appears that synapses with a larger vesicle pool also tend to have a higher release probability (Dobrunz and Stevens, 1997). Hence facilitation is expected to be more dominant at “smaller” synapses.

This extension of the depletion model can account quite well for data where the simpler depletion model fails, in particular the relationship between stimulus frequency and steady-state response amplitude (Varela et al., 1997; Markram et al., 1998). For instance, a comprehensive survey of cells in the medial prefrontal cortex has shown that this model can fit a wide range of different behaviors encountered in such data sets, despite large variability in the relative contribution of depression and facilitation (Wang et al., 2006).

This depletion model with facilitation has become very popular as a canonical model for short term plasticity. It has, either in the form given here (Equations 3, 4) or using a slightly different set of equations as introduced by Tsodyks et al. (1998), been used in many studies investigating the functional importance of short term plasticity (see e.g., Abbott et al., 1997; Tsodyks et al., 1998; Fuhrmann et al., 2002; Mongillo et al., 2008; Pfister et al., 2010). As usual, however, a closer experimental investigation of synapses has shown that this relatively simple and intuitive model lacks potentially important detail, as will be discussed in the following sections.

## Use-dependent vesicle replenishment

An important observation at odds with the depletion model is that vesicle replenishment can accelerate after intensive stimulation. This effect was found to depend on an increase in intracellular calcium concentration, and to occur in a physiological range of input firing rates (Dittman and Regehr, 1998; Stevens and Wesseling, 1998; Wang and Kaczmarek, 1998; Sakaba and Neher, 2001; Fuhrmann et al., 2004; Hosoi et al., 2007). Enhanced vesicle replenishment can be included in the depletion model by adding some form of activity-dependent component to Equation (3). Two slightly different approaches have been proposed, both capable of explaining the slow reduction in steady state depression for strong stimuli that the simple depletion model fails to replicate.

The first model, introduced by Fuhrmann et al. (2004) to reproduce depression at cortical synapses, was based on the idea that presynaptic activity directly modulates the time constant τ_*r*_ of vesicle replenishment in Equation (3) above:

(5) $$\frac{d\tau_{r}(t)}{dt}=\frac{\tau_{r0}-\tau_{r}(t)}{\tau_{\text{FDR}}}-a_{\text{FDR}}\tau_{r}(t)\;\cdot \;\sum_{j}\delta (t-t_{j})$$

Here each presynaptic action potential reduces the time constant by *a*_FDR_τ_*r*_(*t*), which recovers to its resting value τ_*r*0_ with a time constant τ_FDR_ in the order of hundreds of milliseconds. A very similar model with a non-linear relation between intracellular calcium concentration and recovery rate was proposed to explain the different kinetics observed at hippocampal and cerebellar synapses (Dittman and Regehr, 1998; Dittman et al., 2000).

Alternatively, it may be assumed activity leads to a temporary enhancement of vesicle replenishment. This is based on the observation that high-frequency stimulation causes a fast but short-lived component of recovery from depression, which is absent after weaker stimulation (Wang and Kaczmarek, 1998). In these experiments, the recovery time course was fit by two exponential functions, suggesting the combined action of at least two processes. This can be modeled by augmenting a constant background replenishment with a low rate $\left(k_{r}=\frac{1}{\tau_{r}}\right)$ with an activity-dependent component:

(6) $$\frac{dk_{e}(t)}{dt}=-\frac{k_{e}(t)}{\tau_{e}}+a_{e}\;\cdot \;\sum_{j}\delta (t-t_{j})\;\cdot \;(1-k_{e}(t))$$

This process is activated by presynaptic activity, leads to an increment *a*_*e*_ of the replenishment rate for each action potential, and decays with a time constant τ_*e*_ in the range of 10–100 ms. Equation (3) then becomes:

(7) $$\frac{dn(t)}{dt}=\underset{r\text{replenishment}}{\underset{︸}{\left(k_{r}+\tilde{k_{e}}k_{e}(t))(1-n(t)\right)}}\underset{\text{release}}{\underset{︸}{-\sum_{j}\delta (t-t_{j})\;\cdot \;p(t)\;\cdot \;n(t)}}$$

where ${\tilde{k}}_{e}$ is the peak rate of activity-dependent vesicle replenishment. This model predicts weaker steady-state depression at high frequencies (Figures 2C,F), and has been shown to rather accurately reproduce the vesicle pool kinetics (Hosoi et al., 2007) and steady-state behavior at the calyx of Held (Wong et al., 2003; Hennig et al., 2008).

The biophysical mechanism behind use-dependent vesicle replenishment is still not well understood. It appears clear that it depends on calcium influx (Wang and Kaczmarek, 1998; Sakaba and Neher, 2001; Hosoi et al., 2007), but it has been difficult to experimentally disentangle the role of calcium-dependent vesicle recruitment and calcium-dependent endocytosis, perhaps because most studies so far used extremely strong and unphysiological stimuli to deplete the vesicle pool. A recent study suggests that these two processes may in fact be linked, and that perhaps the speed at which release sites are made available by endocytosis is an important rate limiting step during high frequency transmission (Yao and Sakaba, 2012). Use-dependent replenishment may then reflect faster recruitment due to more efficient endocytosis.

A main function of this mechanism appears to maintain the ability of a synapse to transmit during sustained periods of high activity (Wong et al., 2003; Hosoi et al., 2007). It is as such an important, and often overlooked component of short term plasticity that has implications for transmission of varying firing rates. In addition, it has been suggested to improve transmission by broadening the range over which information about rate and rate changes are reliably transmitted (Fuhrmann et al., 2004; Yang et al., 2009). Which of the two models discussed here is more appropriate is unclear. The difference between the two models is that enhanced replenishment is unbounded in Equation (5), but bounded in Equation (6). Hence the former predicts a faster decrease of the steady state response amplitude with increasing frequency, which more quickly settles to a constant value. It is therefore possible that it underestimates the amount of depression at some synapses, but this would require a more exhaustive comparison with data.

## Slow modulation of release probability

A further omission of the depletion model is that activity-dependent release probability suppression may also contribute to synaptic depression (Xu and Wu, 2005; Mochida et al., 2008). Potential mechanisms include VGCC inactivation (Forsythe et al., 1998; Patil et al., 1998) or activation of presynaptic autoreceptors such as mGluRs or AMPARs, which in turn can cause a reduction of the release probability (Takahashi et al., 1996; Takago et al., 2005). A possible molecular route of such effects is calcium/calmodulin (Lee et al., 1999). Postsynaptic release of endocannabinoids has also been shown to suppress synaptic strength over short time scales, but the mechanisms are currently not well understood (Brenowitz and Regehr, 2005). Overall, the degree to which these mechanisms are relevant under physiological conditions is still not fully understood. For instance, release probability suppression has been reported to strongly contribute to synaptic depression during weak activity at the calyx of Held (Xu and Wu, 2005), but this effect may be more pronounced at immature synapses were morphological development renders synaptic transmission is less effective (Renden et al., 2005; Nakamura et al., 2008).

A generic model incorporating both release probability facilitation and depression can be constructed by extending Equation (4) by an activity-dependent modulation of the baseline release probability *p*_0_ (Billups et al., 2005; Hennig et al., 2008):

(8) $$\frac{dp_{0}(t)}{dt}=-\frac{{\tilde{p}}_{0}-p_{0}(t)}{\tau_{i}}-\sum_{j}\delta (t-t_{j})\;\cdot \;a_{i}\;\cdot \;p_{0}(t)$$

Here the baseline release probability *p*_0_(*t*) is reduced by a constant fraction *a*_*i*_ after each spike, and recovers back to ${\tilde{p}}_{0}$ with a time constant τ_*i*_ in the order of several seconds. Then depression of release probability is proportional to the incoming spike rate. An alternative form, which models the activation of autoreceptors, is to replace the term on the right-hand side with ∑_*j*_ δ(*t* − *t*_*j*_) · *a*_*a*_ · *p*_0_(*t*) · *p*(*t*) · *n*(*t*). In this case, depression of release probability is release-dependent. Combinations of both mechanisms are also possible, as shown by Hennig et al. (2008). In combination with the depletion model and facilitation (Equations 3 or 6, and Equation 4), this model can account for a slow form of depression that follows initial rapid vesicle depletion (Figures 2D,F), as observed at GABAergic synapses (Kraushaar and Jonas, 2000) or the calyx of Held (Hennig et al., 2008) during prolonged stimulation.

The analysis of the steady-state behavior the model reveals an interesting further property (Hennig et al., 2007). If the release probability is assumed to vary slowly compared to the effective vesicle replenishment rate ${\tilde{k}}_{e}$, the quasi-stationary solution of Equation (3) with use-dependent vesicle replenishment (Equation 6) is $n_{\infty }p_{c}={\tilde{k}}_{e}(1-n_{\infty })$, where the index *c* indicates that *p*_*c*_ is constant over the time interval considered, and we obtain *n*_∞_ = *k*_*e*_/(*p*_*c*_ + *k*_*e*_). This solution is valid when all fast processes (e.g., facilitation) have settled to their stationary values. If the release probability is now changed by a small amount to *p*′_*c*_ = α*p*_*c*_, then the vesicle pool occupancy settles to a value that differs by a factor of *n*′_∞_/*n*_∞_ = (*p*_*c*_ + *k*_*e*_)/(α*p*_*c*_ + *k*_*e*_).

Hence a slow reduction in release probability will not only slowly depress the postsynaptic response, but also increase the size of the releasable vesicle pool (Figure 2G). This corresponds to a transfer of depression from vesicle depletion to a reduction of release probability. The net effect is a decrease in postsynaptic response that is slower than the change in release probability, and a concomitant refilling of the vesicle pool. Analysis of synaptic depression at the calyx of Held during prolonged stimulation support this conclusion, and suggest that it is, in part, mediated by mGluR autoreceptor activation (Billups et al., 2005; Hennig et al., 2008).

### A closer look at release probability

A central variable in the models discussed is the release probability, and so far the effect of activity was assumed to be linear. This is however, incompatible with the steep non-linearity that couples presynaptic calcium influx to release rate (Bollmann et al., 2000; Schneggenburger and Neher, 2000; Lou et al., 2005). If we assume that the effects of facilitation and depression discussed above such as accumulation of residual calcium, channel facilitation or inactivation, have a linear effect on the calcium concentration, this non-linearity would predict a far more drastic effect on the release rate. In fact, early studies already found that a third to fourth-power relationship is a better model for facilitation than a linear model (Zengel and Magleby, 1982).

An analysis of synaptic depression at the calyx of Held by Xu and Wu (2005) further confirms this intuition. This study suggested that depression during slow stimulation (in the range between 1 and 10 Hz) is primarily mediated by a reduction in calcium influx, while vesicle depletion is only effective at higher frequencies. Interestingly the model presented in the preceding section qualitatively reproduces this effect. As shown in Figure 2F, slow depression of release probability has a significant effect at low frequencies when compared to an equivalent depletion model, which becomes weaker with increasing frequency. However, as shown above this model also predicts that the depression at higher frequencies is still due to reduced release probability, which replaces vesicle depletion during sustained activity. There is some experimental evidence based on fluctuation analysis in support of this hypothesis (Hennig et al., 2008), but it will be interesting to see if alternative vesicle depletion models can also account for these findings.

### Augmentation and post-tetanic potentiation

Augmentation and post-tetanic potentiation are two slowly developing and long-lasting forms of synaptic enhancement (Fisher, 1997; Zucker and Regehr, 2002). They are induced by prolonged stimulation of the synapse, and vary in their activation and relaxation kinetics. The faster form, with time constants of seconds, is typically referred to as augmentation, whereas post-tetanic potentiation operates on time scales of tens of seconds. It appears that these processes are caused by an increase in release probability, which can be occluded by depression due to vesicle depletion during ongoing stimulation (Habets and Borst, 2007). While early studies proposed accumulation of residual calcium at the synaptic terminal as a primary mechanism (Zengel and Magleby, 1982; Zucker and Lara-Estrella, 1983; Habets and Borst, 2007), more recent work also implicated PKC activation (Korogod et al., 2007) or calmodulin/CaM kinase II activity (Fiumara et al., 2007).

A model which could account for a range of findings in data from the frog and toad neuromuscular junction was proposed by Zengel and Magleby (1982). They proposed that facilitation (*F*), augmentation (*A*) and post-tetanic potentiation (*P*) affect release probability in a multiplicative manner:

(9) P(t)∝F(t)A(t)P(t)

Each process follows first order kinetics, and facilitation was best captured by including a fast and a slow component (see also Zucker and Lara-Estrella, 1983). While facilitation required a fourth-power relationship between the corresponding state variables and release rate, it was sufficient to assume a linear dependence for augmentation and potentiation. This points to different potential sites of action of these mechanisms as outlined above. In addition, it was found that augmentation increases with longer stimuli. This was modeled by including a time-dependent increase in activation rate *a* = *a*_0_*z*^ν*T*^ (where ν is the stimulus frequency, *T* is stimulus time and *z* a constant), but could also indicate the presence of multiple first-order processes acting on different time scales (Drew and Abbott, 2006; Hennig et al., 2008). For instance, activation of presynaptic NMDA receptors has also been shown to enhance release probability, with a time course in the order of minutes (Duguid and Smart, 2004).

So far, few theoretical studies have investigated the implications of slow enhancement of release using detailed models. A simple, phenomenological model based on Equation (4) above, where time constants were chosen in the range of augmentation, suggests a potential role in short term memory (Mongillo et al., 2008).

## The other side: receptor desensitization

The time course of the postsynaptic response depends not only on the amount of released transmitter and its time course, but also on the kinetics of the receptors. The interplay of these factors with synapse morphology has been investigated in great detail with Monte Carlo simulations (Stiles et al., 1996; Franks et al., 2003; Coggan et al., 2005; Postlethwaite et al., 2007), which are in particular useful to understand the sources of variability at synapses. The semi-quantitative models discussed in this review cannot easily accommodate this level of detail, but can still be extended to include salient aspects of the postsynaptic response (Destexhe et al., 1994a; Roth and Rossum, 2009).

Apart from the response latency and duration, desensitization is an important property of receptors which has been shown to contribute to synaptic depression during physiological activity levels (Trussell et al., 1988, 1993; Jones and Westbrook, 1996; Neher and Sakaba, 2001). A simple but effective approximation of the state of the population of receptors *D*(*t*), can be modeled using first order kinetics:

(10) $$\frac{dD(t)}{dt}=\frac{1-D(t)}{\tau_{D}}-\sum_{j}\delta (t-t_{j})\;\cdot \;a_{D}\;\cdot \;p(t)\;\cdot \;n(t)\;\cdot \;D(t)$$

The quantity *D*(*t*) represents the fraction of non-desensitized receptors. Recovery from desensitization τ_*D*_ is typically in the order of tens of milliseconds, such that it is only effective during intense episodes of activity. The postsynaptic response is then expressed as *R*(*t*) = *g*_*m*_*D*(*t*) · *n*(*t*) · *p*(*t*), where *g*_*m*_ is the peak conductance.

This basic model captures synaptic depression due to desensitization well. In particular, simulations have shown that a main effect is the masking of presynaptic facilitation at high stimulus frequencies (Jones and Westbrook, 1996; Wong et al., 2003). Yet in this form the model obviously neglects the time course of the postsynaptic potential, which can also be affected by desensitization. To model this, it is possible to extend it by adding more states, such as closed, open and desensitized, and to model state transitions in a transmitter concentration-dependent manner as a Markov process. Such models have been proposed to better account for the kinetics of the postsynaptic response, in particular for kinetics of different receptor subunit composition (Destexhe et al., 1994a; Robert et al., 2005; Postlethwaite et al., 2007). A drawback of this approach is that this also requires an appropriate model of the time course of neurotransmitter seen by the receptors, which has to be obtained by more detailed diffusion models (see e.g., Franks et al., 2003; Postlethwaite et al., 2007). Finally it is also worth mentioning that potentially other postsynaptic mechanisms exist that contribute to short term plasticity, which have not yet been investigated in models. For instance, AMPA receptors can show an increased paired-pulse facilitation during activity-dependent relief of polyamide block (Rozov and Burnashev, 1999). This effect is potentially important at immature synapses lacking the GluR2 AMPA receptor subunit.

## Stochasticity of synapses

Transmitter release is a stochastic process, and as a consequence the magnitude of the postsynaptic current evoked by each presynaptic action potential fluctuates from time to time. Due to the quantal nature of synaptic transmission, the variance of the postsynaptic response is described by binomial statistics, with a predicted variance of Var(*g*(*t*)) = *g*_*m*_ · *N*(*t*) · *p*(*t*) · (1 − *p*(*t*)) (Del Castillo and Katz, 1954). This shows that changes in the synaptic parameters due to short term plasticity will not only cause changes in the average postsynaptic response, but also in the magnitude of the fluctuations, as measured by the coefficient of variation:

(11) $$CV(g(t))=\sqrt{\frac{1-p(t)}{N(t)p(t)g_{m}}}$$

This value is high when the release probability or the number of release-ready vesicles is small, as, for instance, often found for cortical neurons (Wang et al., 2006; Brémaud et al., 2007). The expression also shows that stochastic effects are bound to be more important when synaptic depression is dominated by vesicle depletion. In addition, the entire vesicle cycle, which includes vesicle replenishment, consists of stochastic events. In contrast, the influx of calcium during an action potential, which triggers transmitter release, is considered a much more salient event, and is therefore expected to contribute much less to postsynaptic response variability. To model the main sources of stochasticity of synaptic, the models discussed above can be directly cast into a stochastic form by simulating vesicle release and replenishment as random events (see e.g., de la Rocha and Parga, 2005; Yang et al., 2009; Rosenbaum et al., 2012).

Stochastic models are extremely useful for quantitative evaluation of models of synaptic transmission and plasticity, since postulated changes in *N* and *p* have predictable effects on variability that can be directly tested experimentally (see e.g., Quastel, 1997; Scheuss and Neher, 2001; Brémaud et al., 2007). This type of analysis requires a careful dissection of synaptic function, since for instance conductance changes through receptor desensitization may be mistaken for presynaptic effects if not properly controlled for. Recent work exploiting this approach provided evidence for substantial variability of synaptic parameters for synapses between cortical pyramidal neurons, the presence of multi-quantal release (Loebel et al., 2009), and the coordination of pre-synaptic release probability and postsynaptic synaptic strength (Hardingham et al., 2010).

Studies investigating stochastic synapse models have reported several effects indicating that this also has important implications for neural computations and network function. For instance, shot noise due to stochastic release can increase the output firing rate of a neuron operating in a fluctuation driven regime when compared to deterministic dynamics (de la Rocha and Parga, 2005). The same study also showed that short term depression in stochastic synapses causes a further, non-monotonic modulation of output firing in presence of input correlations (see also Rosenbaum et al., 2012). Such effects were further analyzed by Rosenbaum et al. (2013), who showed that, unlike for a deterministic model, a stochastic synapse with short term depression can significantly de-correlate neural activity. Finally, an analysis of stochastic models including slow release probability modulation and activity-dependent vesicle replenishment suggested that multiple mechanisms of short term plasticity may act synergistically to maintain stable information transmission over a broad range of input frequencies (Yang et al., 2009). Overall, however, the models used so far to analyze stochastic effects were mostly rather simple, typically only the depletion model was considered, and assumed constant random inputs to the neuron (see Merkel and Lindner, 2010, for an extension).

## Outlook

Theoretical models have contributed much to our understanding of synaptic transmission and short term plasticity by providing a framework to express conceptual models in rigorous terms, and to derive quantitatively testable hypotheses. The models discussed here capture the central biophysical processes involved in synaptic transmission in relatively simple mathematical form, such that an exact or at least approximate analytical treatment is possible. Moreover, key variables in these models have direct measurable correlates. This supports analysis and comparison with data, as often exploited for deriving synaptic parameters from experimentally recorded synaptic currents. It is however not straight forward to experimentally interfere with short term plasticity in intact neural circuits in a targeted manner, for instance to assess functional implications and consequences. Therefore, these models are also a valuable tool that enables analysis beyond the experimentally feasible.

The basic depletion model with facilitation has passed the test of time, which nicely illustrates the success of simple, mathematically tractable phenomenological models in biology. However, as shown here, short term plasticity can be more complicated. In particular slow forms of synaptic depression and facilitation merit more thorough investigation, both in terms of mechanisms and their relevance for neural computations. While the depletion model can very successfully replicate even synaptic responses during *in vivo*-like activity patterns (Hermann et al., 2009), slow synaptic modulation may have important effects during firing rate modulation on time scales of tens of seconds (see e.g., Mongillo et al., 2008). A combination of slow facilitation and depression has also been shown to support differential responses to time varying stimuli (Barak and Tsodyks, 2007). These studies show that these mechanisms certainly warrant further investigation.

As shown in this review, even the extended and more complete models of short term plasticity have a relatively simple mathematical form, which will greatly facilitate the understanding of their effects in networks. Perhaps a central question in this context is in how far the different mechanisms discussed here have direct functional implications, or rather reflect the biophysical properties and limitations of chemical signaling between neurons. Some of the studies touched upon above and in the previous section suggest the former may be the case (for a more detailed discussion, see e.g., Abbott and Regehr, 2004). On the other hand it is equally plausible some aspects of short term plasticity may related to homeostatic effects or metabolic efficacy of synapses, issues that have received little attention so far and are now easily testable in models. Addressing such questions may require the analysis of the models under more physiologically relevant conditions. For example, recent experiments indicate that unreliable synapses with short term plasticity are particularly suited to transmit information contained in brief bursts of activity typically observed in hippocampus (Rotman et al., 2011). Therefore, modeling studies specifically investigating synapses in their “natural habitat” of recurrent networks should allow us to refine and consolidate such hypotheses, and to establish more of the much sought-after links between neural biophysics and brain function and dysfunction.

### Conflict of interest statement

The author declares that the research was conducted in the absence of any commercial or financial relationships that could be construed as a potential conflict of interest.

## References

[B1] Abbott L. F. Regehr W. G. (2004). Synaptic computation. Nature 431, 796–803 10.1038/nature03010 15483601

[B2] Abbott L. F. Varela J. A. Sen K. and Nelson S. B. (1997). Synaptic depression and cortical gain control. Science 275, 220–224 8985017 10.1126/science.275.5297.221

[B3] Atluri P. P. Regehr W. G. (1996). Determinants of the time course of facilitation at the granule cell to Purkinje cell synapse. J. Neurosci. 16, 5661–5671 8795622 10.1523/JNEUROSCI.16-18-05661.1996 PMC6578977

[B4] Barak O. Tsodyks M. (2007). Persistent activity in neural networks with dynamic synapses. PLoS Comput. Biol. 3:e35 10.1371/journal.pcbi.0030035 17319739 PMC1808024

[B5] Betz W. J. (1970). Depression of transmitter release at the neuromuscular junction of the frog. J. Physiol. 206, 629–644 5498509 10.1113/jphysiol.1970.sp009034 PMC1348669

[B6] Billups B. Graham B. P. Wong A. Y. C. Forsythe I. D. (2005). Unmasking group III metabotropic glutamate autoreceptor function at excitatory synapses in the rat CNS. J. Physiol. 565, 885–896 10.1113/jphysiol.2005.086736 15845577 PMC1464548

[B7] Blatow M. Caputi A. Burnashev N. Monyer H. and Rozov A. (2003). Ca2+ buffer saturation underlies paired pulse facilitation in calbindin-D28k-containing terminals. Neuron 38, 79–88 10.1016/S0896-6273(03)00196-X 12691666

[B8] Bollmann J. H. Sakmann B. and Borst J. G. (2000). Calcium sensitivity of glutamate release in a calyx-type terminal. Science 289, 953–957 10.1126/science.289.5481.953 10937999

[B9] Borst J. G. Sakmann B. (1998). Calcium current during a single action potential in a large presynaptic terminal of the rat brainstem. J. Physiol. 506, 143–157 10.1111/j.1469-7793.1998.143bx.x 9481678 PMC2230710

[B10] Branco T. Staras K. (2009). The probability of neurotransmitter release: variability and feedback control at single synapses. Nat. Rev. Neurosci. 10, 373–383 10.1038/nrn2634 19377502

[B11] Brémaud A. West D. C. Thomson A. M. (2007). Binomial parameters differ across neocortical layers and with different classes of connections in adult rat and cat neocortex. PNAS 104, 14134–14139 10.1073/pnas.0705661104 17702864 PMC1949494

[B12] Brenowitz S. D. Regehr W. G. (2005). Associative short-term synaptic plasticity mediated by endocannabinoids. Neuron 45, 419–431 10.1016/j.neuron.2004.12.045 15694328

[B13] Coggan J. S. Bartol T. M. Esquenazi E. Stiles J. R. Lamont S. Martone M. E. (2005). Evidence for ectopic neurotransmission at a neuronal synapse. Science 309, 446–451 10.1126/science.1108239 16020730 PMC2915764

[B14] Cuttle M. F. Tsujimoto T. Forsythe I. D. Takahashi T. (1998). Facilitation of the presynaptic calcium current at an auditory synapse in rat brainstem. J. Physiol. 512, 723–729 10.1111/j.1469-7793.1998.723bd.x 9769416 PMC2231247

[B15] de la Rocha J. Parga N. (2005). Short-term synaptic depression causes a non-monotonic response to correlated stimuli. J. Neurosci. 25, 8416–8431 10.1523/JNEUROSCI.0631-05.2005 16162924 PMC6725676

[B16] De Robertis E. D. Bennett H. S. (1955). Some features of the submicroscopic morphology of synapses in frog and earthworm. J. Biochem. Cytol. 1, 47–58 14381427 10.1083/jcb.1.1.47 PMC2223594

[B17] Del Castillo J. Katz B. (1954). Quantal components of the end-plate potential. J. Physiol. 124, 560–573 13175199 10.1113/jphysiol.1954.sp005129 PMC1366292

[B18] Destexhe A. Mainen Z. F. Sejnowski T. J. (1994a). An efficient method for computing synaptic conductances based on a kinetic model of receptor binding. Neural Comput. 6, 14–18

[B19] Destexhe A. Mainen Z. F. Sejnowski T. J. (1994b). Synthesis of models for excitable membranes, synaptic transmission and neuromodulation using a common kinetic formalism. J. Comput. Neurosci. 1, 195–231 8792231 10.1007/BF00961734

[B20] Dittman J. S. Kreitzer A. C. Regehr W. G. (2000). Interplay between facilitation, depression, and residual calcium at three presynaptic terminals. J. Neurosci. 20, 1374–1385 10662828 10.1523/JNEUROSCI.20-04-01374.2000 PMC6772383

[B21] Dittman J. S. Regehr W. G. (1998). Calcium dependence and recovery kinetics of presynaptic depression at the climbing fiber to Purkinje cell synapse. J. Neurosci. 18, 6147–6162 9698309 10.1523/JNEUROSCI.18-16-06147.1998 PMC6793194

[B22] Dobrunz L. E. Stevens C. F. (1997). Heterogeneity of release probability, facilitation, and depletion at central synapses. Neuron 18, 995–1008 9208866 10.1016/s0896-6273(00)80338-4

[B23] Drew P. J. Abbott L. F. (2006). Models and properties of power-law adaptation in neural systems. J. Neurophysiol. 96, 826–833 10.1152/jn.00134.2006 16641386

[B24] Duguid I. C. Smart T. G. (2004). Retrograde activation of presynaptic NMDA receptors enhances GABA release at cerebellar interneuron-Purkinje cell synapses. Nat. Neurosci. 7, 525–533 10.1038/nn1227 15097992

[B25] Felmy F. Neher E. Schneggenburger R. (2003). Probing the intracellular calcium sensitivity of transmitter release during synaptic facilitation. Neuron 37, 801–811 10.1016/S0896-6273(03)00085-0 12628170

[B26] Fioravante D. Regehr W. G. (2011). Short-term forms of presynaptic plasticity. Curr. Opin. Neurobiol. 21, 269–274 10.1016/j.conb.2011.02.003 21353526 PMC3599780

[B27] Fisher S. (1997). Multiple overlapping processes underlying short-term synaptic enhancement. Trends Neurosci. 20, 170–177 10.1016/S0166-2236(96)01001-6 9106358

[B28] Fiumara F. Milanese C. Corradi A. Giovedi S. Leitinger G. Menegon A. (2007). Phosphorylation of synapsin domain A is required for post-tetanic potentiation. J. Cell. Sci. 120, 3321. 10.1242/jcs.012005 17726061 PMC3016615

[B29] Forsythe I. D. Tsujimoto T. Barnes-Davies M. Cuttle M. F. Takahashi T. (1998). Inactivation of presynaptic calcium current contributes to synaptic depression at a fast central synapse. Neuron 20, 797–807 10.1016/S0896-6273(00)81017-X 9581770

[B30] Franks K. M. Stevens C. F. Sejnowski T. J. (2003). Independent sources of quantal variability at single glutamatergic synapses. J. Neurosci. 23, 3186–3195 12716926 10.1523/JNEUROSCI.23-08-03186.2003 PMC2944019

[B31] Fuhrmann G. Cowan A. Segev I. Tsodyks M. Stricker C. (2004). Multiple mechanisms govern the dynamics of depression at neocortical synapses of young rats. J. Physiol. 557, 415–438 10.1113/jphysiol.2003.058107 15020700 PMC1665093

[B32] Fuhrmann G. Segev I. Markram H. Tsodyks M. (2002). Coding of temporal information by activity-dependent synapses. J. Neurophysiol. 87, 140–148 11784736 10.1152/jn.00258.2001

[B33] Habets R. L. P. Borst J. G. G. (2007). Dynamics of the readily releasable pool during post-tetanic potentiation in the rat calyx of Held synapse. J. Physiol. 581, 467–478 10.1113/jphysiol.2006.127365 17363387 PMC2075193

[B34] Hardingham N. R. Read J. C. A. Trevelyan A. J. Nelson J. C. Jack J. J. B. Bannister N. J. (2010). Quantal analysis reveals a functional correlation between presynaptic and postsynaptic efficacy in excitatory connections from rat neocortex. J. Neurosci. 30, 1441–1451 10.1523/JNEUROSCI.3244-09.2010 20107071 PMC2825095

[B35] Hennig M. H. Postlethwaite M. Forsythe I. D. Graham B. P. (2007). A biophysical model of short-term plasticity at the calyx of Held. Neurocomputing 70, 1626–1629

[B36] Hennig M. H. Postlethwaite M. Forsythe I. D. Graham B. P. (2008). Interactions between multiple sources of short-term plasticity during evoked and spontaneous activity at the rat calyx of Held. J. Physiol. 586, 3129–3146 10.1113/jphysiol.2008.152124 18450780 PMC2538789

[B37] Hermann J. Grothe B. Klug A. (2009). Modeling short-term synaptic plasticity at the calyx of held using *in vivo*-like stimulation patterns. J. Neurophysiol. 101, 20–30 10.1152/jn.90243.2008 18971300

[B38] Hosoi N. Sakaba T. Neher E. (2007). Quantitative analysis of calcium-dependent vesicle recruitment and its functional role at the calyx of Held synapse. J. Neurosci. 27, 14286–14298 10.1523/JNEUROSCI.4122-07.2007 18160636 PMC6673456

[B39] Jones M. V. Westbrook G. L. (1996). The impact of receptor desensitization on fast synaptic transmission. Trends Neurosci. 19, 96–101 10.1016/S0166-2236(96)80037-3 9054063

[B40] Katz B. Miledi R. (1968). The role of calcium in neuromuscular facilitation. J. Physiol. 195, 481–492 4296699 10.1113/jphysiol.1968.sp008469 PMC1351674

[B41] Korogod N. Lou X. Schneggenburger R. (2007). Posttetanic potentiation critically depends on an enhanced Ca(2+) sensitivity of vesicle fusion mediated by presynaptic PKC. PNAS 104, 15923–15928 10.1073/pnas.0704603104 17884983 PMC2000442

[B42] Kraushaar U. Jonas P. (2000). Efficacy and stability of quantal GABA release at a hippocampal interneuron-principal neuron synapse. J. Neurosci. 20, 5594–5607 10908596 10.1523/JNEUROSCI.20-15-05594.2000 PMC6772523

[B43] Lee A. Wong S. T. Gallagher D. Li B. Storm D. R. Scheuer T. Catterall W. A. (1999). Ca2+/calmodulin binds to and modulates P/Q-type calcium channels. Nature 399, 155–159 10.1038/20194 10335845

[B44] Liley A. W. North K. A. (1953). An electrical investigation of effects of repetitive stimulation on mammalian neuromuscular junction. J. Neurophysiol. 16, 509–527 13097199 10.1152/jn.1953.16.5.509

[B45] Loebel A. Silberberg G. Helbig D. Markram H. Tsodyks M. Richardson M. J. E. (2009). Multiquantal release underlies the distribution of synaptic efficacies in the neocortex. Front. Comput. Neurosci. 3:27 10.3389/neuro.10.027.2009 19956403 PMC2786302

[B46] Lou X. Scheuss V. Schneggenburger R. (2005). Allosteric modulation of the presynaptic Ca2+ sensor for vesicle fusion. Nature 435, 497–501 10.1038/nature03568 15917809

[B47] Markram H. Wang Y. Tsodyks M. (1998). Differential signaling via the same axon of neocortical pyramidal neurons. PNAS 95, 5323–5328 9560274 10.1073/pnas.95.9.5323 PMC20259

[B48] Merkel M. Lindner B. (2010). Synaptic filtering of rate-coded information. Phys. Rev. E 81:041921 10.1103/PhysRevE.81.041921 20481767

[B49] Mochida S. Few A. P. Scheuer T. Catterall W. A. (2008). Regulation of presynaptic Ca(V)2.1 channels by Ca2+ sensor proteins mediates short-term synaptic plasticity. Neuron 57, 210–216 10.1016/j.neuron.2007.11.036 18215619

[B50] Mongillo G. Barak O. Tsodyks M. (2008). Synaptic theory of working memory. Science 319, 1543–1546 10.1126/science.1150769 18339943

[B51] Morris R. G. M. (2003). Long-term potentiation and memory. Philos. Trans. R. Soc. Lond. Ser. B 358, 643–647 10.1098/rstb.2002.1230 12740109 PMC1693171

[B52] Nakamura T. Yamashita T. Saitoh N. Takahashi T. (2008). Developmental changes in cal-cium/calmodulin-dependent inacti-vation of calcium currents at the rat calyx of Held. J. Physiol. 586, 2253–2261 10.1113/jphysiol.2007.142521 18238813 PMC2479563

[B53] Neher E. Sakaba T. (2001). Combining deconvolution and noise analysis for the estimation of transmitter release rates at the calyx of held. J. Neurosci. 21, 444–461 11160425 10.1523/JNEUROSCI.21-02-00444.2001 PMC6763797

[B54] Patil P. G. Brody D. L. Yue D. T. (1998). Preferential closed-state inactivation of neuronal calcium channels. Neuron 20, 1027–1038 10.1016/S0896-6273(00)80483-3 9620706

[B55] Pfister J.-P. Dayan P. Lengyel M. (2010). Synapses with short-term plasticity are optimal estimators of presynaptic membrane potentials. Nat. Neurosci. 13, 1271–1275 10.1038/nn.2640 20852625 PMC3558743

[B56] Postlethwaite M. Hennig M. H. Steinert J. R. Graham B. P. Forsythe I. D. (2007). Acceleration of AMPA receptor kinetics underlies temperature-dependent changes in synaptic strength at the rat calyx of Held. J. Physiol. 579, 69–84 10.1113/jphysiol.2006.123612 17138605 PMC2075387

[B57] Quastel D. M. (1997). The binomial model in fluctuation analysis of quantal neurotransmitter release. Biophys. J. 72, 728–753 9017200 10.1016/s0006-3495(97)78709-5 PMC1185598

[B58] Renden R. Taschenberger H. Puente N. Rusakov D. A. Duvoisin R. Wang L. Y. (2005). Glutamate transporter studies reveal the pruning of metabotropic glutamate receptors and absence of AMPA receptor desensitization at mature calyx of held synapses. J. Neurosci. 25, 8482–8497 10.1523/JNEUROSCI.1848-05.2005 16162930 PMC3375655

[B59] Rizzoli S. O. Betz W. J. (2005). Synaptic vesicle pools. Nat. Rev. Neurosci. 6, 57–69 10.1038/nrn1583 15611727

[B60] Robert A. Armstrong N. Gouaux J. E. Howe J. R. (2005). AMPA receptor binding cleft mutations that alter affinity, efficacy, and recovery from desensitization. J. Neurosci. 25, 3752–3762 10.1523/JNEUROSCI.0188-05.2005 15829627 PMC6724928

[B61] Rosenbaum R. Rubin J. Doiron B. (2012). Short term synaptic depression imposes a frequency dependent filter on synaptic information transfer. PLoS Comput. Biol. 8:e1002557 10.1371/journal.pcbi.1002557 22737062 PMC3380957

[B62] Rosenbaum R. Rubin J. E. Doiron B. (2013). Short-term synaptic depression and stochastic vesicle dynamics reduce and shape neuronal correlations. J. Neurophysiol. 109, 475–484 10.1152/jn.00733.2012 23114215 PMC3545461

[B63] Roth A. Rossum M. C. W. V. (2009). Modeling synapses, in Computational Modeling Methods for Neuroscientists. Vol. 06, Chapter Modelings, ed De Schutter E. (Cambridge, MA: MIT Press), 139–160

[B64] Rotman Z. Deng P.-Y. Klyachko V. A. (2011). Short-term plasticity optimizes synaptic information transmission. J. Neurosci. 31, 14800–14809 10.1523/JNEUROSCI.3231-11.2011 21994397 PMC6703406

[B65] Rozov A. Burnashev N. (1999). Polyamine-dependent facilitation of postsynaptic AMPA receptors counteracts paired-pulse depression. Nature 401, 594–598 10.1038/44151 10524627

[B66] Sakaba T. Neher E. (2001). Quantitative relationship between transmitter release and calcium current at the calyx of held synapse. J. Neurosci. 21, 462–476 11160426 10.1523/JNEUROSCI.21-02-00462.2001 PMC6763832

[B67] Scheuss V. Neher E. (2001). Estimating synaptic parameters from mean, variance, and covariance in trains of synaptic responses. Biophys. J. 81, 1970–1989 10.1016/S0006-3495(01)75848-1 11566771 PMC1301672

[B68] Schneggenburger R. Forsythe I. D. (2006). The calyx of Held. Cell Tissue Res. 326, 311–337 10.1007/s00441-006-0272-7 16896951

[B69] Schneggenburger R. Neher E. (2000). Intracellular calcium dependence of transmitter release rates at a fast central synapse. Nature 406, 889–893 10.1038/35022702 10972290

[B70] Stevens C. F. Wesseling J. F. (1998). Activity-dependent modulation of the rate at which synaptic vesicles become available to undergo exocytosis. Neuron 21, 415–424 10.1016/S0896-6273(00)80550-4 9728922

[B71] Stiles J. R. Van Helden D. Bartol T. M. Salpeter E. E. Salpeter M. M. (1996). Miniature endplate current rise times less than 100 microseconds from improved dual recordings can be modeled with passive acetylcholine diffusion from a synaptic vesicle. PNAS 93, 5747–5752 8650164 10.1073/pnas.93.12.5747 PMC39132

[B72] Sudhof T. C. (2004). The synaptic vesicle cycle. Annu. Rev. Neurosci. 27, 509–547 10.1146/annurev.neuro.26.041002.131412 15217342

[B73] Takago H. Nakamura Y. Takahashi T. (2005). G protein-dependent presynaptic inhibition mediated by AMPA receptors at the calyx of Held. PNAS 102, 7368–7373 10.1073/pnas.0408514102 15878995 PMC1129093

[B74] Takahashi T. Forsythe I. D. Tsujimoto T. Barnes-Davies M. and Onodera K. (1996). Presynaptic calcium current modulation by a metabotropic glutamate receptor. Science 274, 594–597 10.1126/science.274.5287.594 8849448

[B75] Trommershäuser J. Schneggenburger R. Zippelius A. Neher E. (2003). Heterogeneous presynaptic release probabilities: functional relevance for short-term plasticity. Biophys. J. 84, 1563–1579 10.1016/S0006-3495(03)74967-4 12609861 PMC1302728

[B76] Trussell L. O. Thio L. L. Zorumski C. F. Fischbach G. D. (1988). Rapid desensitization of glutamate receptors in vertebrate central neurons. PNAS 85, 2834–2838 2895929 10.1073/pnas.85.8.2834 PMC280094

[B77] Trussell L. O. Zhang S. and Raman I. M. (1993). Desensitization of AMPA receptors upon multiquantal neurotransmitter release. Neuron 10, 1185–1196 10.1016/0896-6273(93)90066-Z 7686382

[B78] Tsodyks M. Pawelzik K. Markram H. (1998). Neural networks with dynamic synapses. Neural Comput. 10, 821–835 9573407 10.1162/089976698300017502

[B79] Tsodyks M. V. Markram H. (1997). The neural code between neocortical pyramidal neurons depends on neurotransmitter release probability. PNAS 94, 719–723 9012851 10.1073/pnas.94.2.719 PMC19580

[B80] Turrigiano G. G. Nelson S. B. (2004). Homeostatic plasticity in the developing nervous system. Nat. Rev. Neurosci. 5, 97–107 10.1038/nrn1327 14735113

[B81] Varela J. A. Sen K. Gibson J. Fost J. Abbott L. F. Nelson S. B. (1997). A quantitative description of short-term plasticity at excitatory synapses in layer 2/3 of rat primary visual cortex. J. Neurosci. 17, 7926–7940 9315911 10.1523/JNEUROSCI.17-20-07926.1997 PMC6793910

[B82] Wadel K. Neher E. Sakaba T. (2007). The coupling between synaptic vesicles and Ca2+ channels determines fast neurotransmitter release. Neuron 53, 563–575 10.1016/j.neuron.2007.01.021 17296557

[B83] Wang L. Y. Kaczmarek L. K. (1998). High-frequency firing helps replenish the readily releasable pool of synaptic vesicles. Nature 394, 384–388 10.1038/28645 9690475

[B84] Wang Y. Markram H. Goodman P. H. Berger T. K. Ma J. Goldman-Rakic P. S. (2006). Heterogeneity in the pyramidal network of the medial prefrontal cortex. Nat. Neurosci. 9, 534–542 10.1038/nn1670 16547512

[B85] Wölfel M. Lou X. Schneggenburger R. (2007). A mechanism intrinsic to the vesicle fusion machinery determines fast and slow transmitter release at a large CNS synapse. J. Neurosci. 27, 3198–3210 10.1523/JNEUROSCI.4471-06.2007 17376981 PMC6672471

[B86] Wong A. Y. C. Graham B. P. Billups B. and Forsythe I. D. (2003). Distinguishing between presynaptic and postsynaptic mechanisms of short-term depression during action potential trains. J. Neurosci. 23, 4868–4877 12832509 10.1523/JNEUROSCI.23-12-04868.2003 PMC6741172

[B87] Xu J. Wu L.-G. (2005). The decrease in the presynaptic calcium current is a major cause of short-term depression at a calyx-type synapse. Neuron 46, 633–645 10.1016/j.neuron.2005.03.024 15944131

[B88] Yang Z. Hennig M. H. Postlethwaite M. Forsythe I. D. Graham B. P. (2009). Wide-band information transmission at the calyx of Held. Neural Comput. 21, 991–1017 10.1162/neco.2008.02-08-714 19018705

[B89] Yao L. Sakaba T. (2012). Activity-dependent modulation of endocytosis by calmodulin at a large central synapse. PNAS 109, 291–296 10.1073/pnas.1100608109 22184217 PMC3252931

[B90] Zengel J. E. Magleby K. L. (1982). Augmentation and facilitation of transmitter release. A quantitative description at the frog neuromuscular junction. J. Gen. Physiol. 80, 583–611 6128372 10.1085/jgp.80.4.583 PMC2228708

[B91] Zucker R. S. Lara-Estrella L. O. (1983). Post-tetanic decay of evoked and spontaneous transmitter release and a residual-calcium model of synaptic facilitation at crayfish neuromuscular junctions. J. Gen. Physiol. 81, 355–372 6132958 10.1085/jgp.81.3.355 PMC2215575

[B92] Zucker R. S. Regehr W. G. (2002). Short-term synaptic plasticity. Annu. Rev. Neurosci. 64, 355–405 10.1146/annurev.physiol.64.092501.114547 11826273

